# Receptor-Binding-Motif-Targeted Sanger Sequencing: a Quick and Cost-Effective Strategy for Molecular Surveillance of SARS-CoV-2 Variants

**DOI:** 10.1128/spectrum.00665-22

**Published:** 2022-05-31

**Authors:** Sankar Prasad Chaki, Melissa M. Kahl-McDonagh, Benjamin W. Neuman, Kurt A. Zuelke

**Affiliations:** a Texas A&M Global Health Research Complex, Division of Research, Texas A&M University, College Station, Texas, USA; b Department of Biology, College of Science, Texas A&M University, College Station, Texas, USA; UNMC

**Keywords:** SARS-CoV-2, receptor-binding motif (RBM), Sanger sequencing, mutations, variant surveillance, Alpha variant, Delta variant, Omicron variant, lineages

## Abstract

Whole-genome sequencing (WGS) is the gold standard for characterizing the severe acute respiratory syndrome coronavirus 2 (SARS-CoV-2) genome and identification of new variants. However, the cost involved and time needed for WGS prevent routine, rapid clinical use. This study aimed to develop a quick and cost-effective surveillance strategy for SARS-CoV-2 variants in saliva and nasal swab samples by spike protein receptor-binding-motif (RBM)-targeted Sanger sequencing. Saliva and nasal swabs prescreened for the presence of the nucleocapsid (N) gene of SARS-CoV-2 were subjected to RBM-specific single-amplicon generation and Sanger sequencing. Sequences were aligned by CLC Sequence Viewer 8, and variants were identified based upon specific mutation signature. Based on this strategy, the present study identified Alpha, Beta/Gamma, Delta, and Omicron variants in a quick and cost-effective manner.

**IMPORTANCE** The coronavirus disease 2019 (COVID-19) pandemic resulted in 427 million infections and 5.9 million deaths globally as of 21 February 2022. SARS-CoV-2, the causative agent of the COVID-19 pandemic, frequently mutates and has developed into variants of major public health concerns. Following the Alpha variant (B.1.1.7) infection wave, the Delta variant (B.1.617.2) became prevalent, and now the recently identified Omicron (B.1.1.529) variant is spreading rapidly and forming BA.1, BA.1.1, BA.2, BA.3, BA.4, and BA.5 lineages of concern. Prompt identification of mutational changes in SARS-CoV-2 variants is challenging but critical to managing the disease spread and vaccine/therapeutic modifications. Considering the cost involved and resource limitation of WGS globally, an RBM-targeted Sanger sequencing strategy is adopted in this study for quick molecular surveillance of SARS-CoV-2 variants.

## INTRODUCTION

The ongoing global pandemic of coronavirus disease 2019 (COVID-19) caused by the severe acute respiratory syndrome coronavirus 2 (SARS-CoV-2) resulted in 5.9 million deaths from December 2019 to February 2022 (1). Frequent viral mutation and new variant formation have delayed the end of the pandemic. The World Health Organization (WHO) and the United States Centers for Disease Control and Prevention (CDC) classified the past and present variants of concern (VOC), demonstrating the frequency of viral mutation (2, 3). Preventive vaccination (4–6) and therapeutic strategies (7) have been effective against past variants of concern. However, the emerging Omicron variant bearing 30 mutations in the spike protein with 15 amino acid substitutions in the receptor-binding domain (RBD) has raised the alarm of reduced vaccine (8) or monoclonal antibody therapy (9, 10) efficacy. Monitoring mutational changes and tracking emerging variants on time are critical to modifying vaccine booster strategies and new therapeutic development.

**FIG 1:**
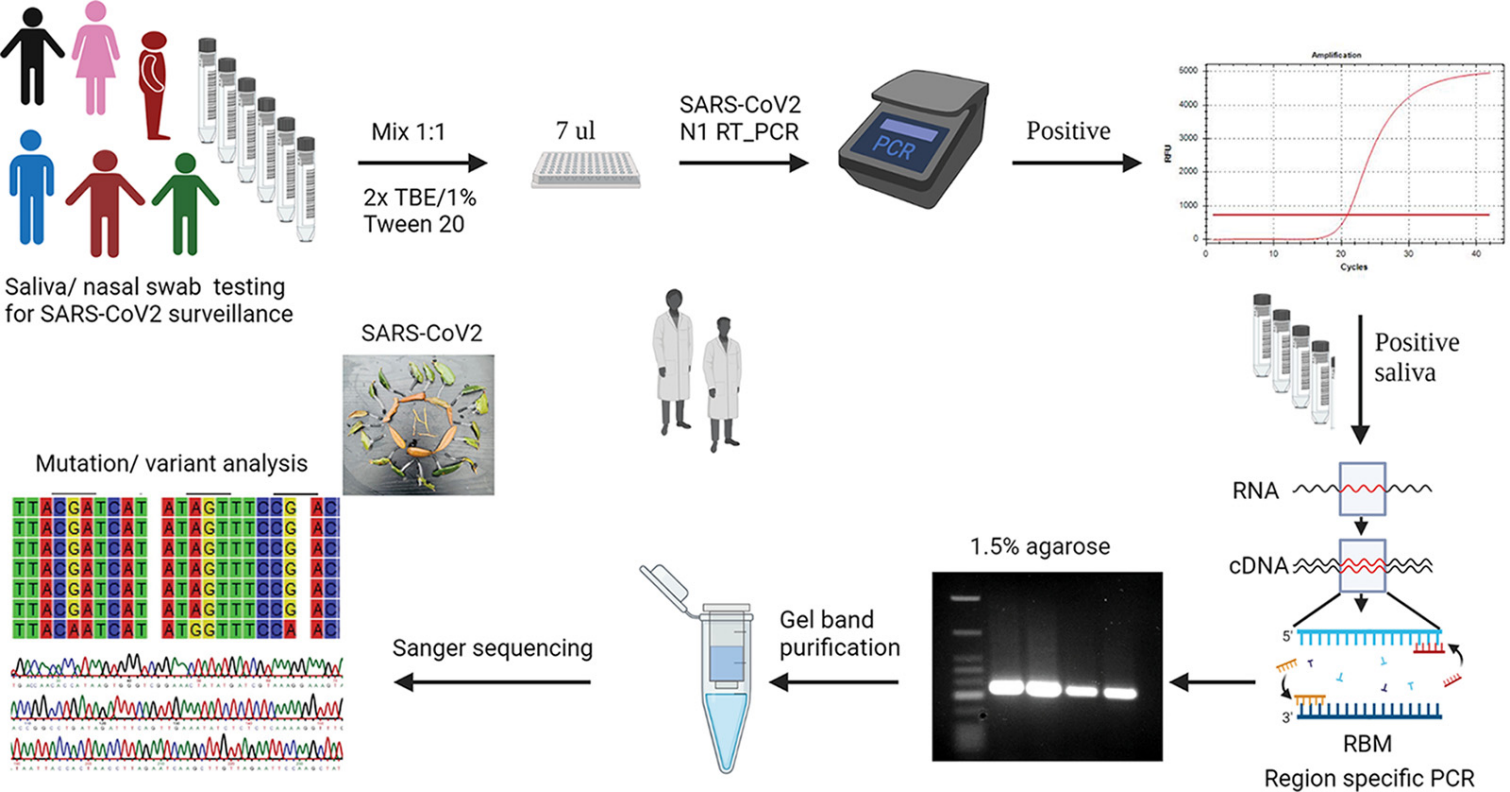
Workflow strategy for quick and cost-effective identification of SARS-CoV-2 variants. The figure shows the initial upstream screening of positive samples in the first step by N gene-targeted RT-qPCR, followed by downstream RNA extraction from positive samples, RBM-targeted amplicon generation, gel band purification, and Sanger sequencing for mutation analysis and variant identifications.

Whole-genome sequencing (WGS) is the gold standard for accurately characterizing new viral genomes and variant designations (11–13). Sanger sequencing was utilized in a few instances for whole-genome sequencing of SARS-CoV-2 (14, 15). Both Sanger sequencing and next-generation sequencing (NGS) were combined to characterize the first whole genome of SARS-CoV-2 from a 2019-nCoV patient sample (16). However, NGS that can generate thousands of reads per sequence in parallel is rapidly adapted in sequencing the whole genome of SARS-CoV-2 and variant identifications (17, 18). In many countries or areas worldwide, lack of instrumentation, reagent facilities, data storage issues, bioinformatics support, and time requirements limit the usage of WGS for routine clinical use or surveillance worldwide. Once the whole genome of a new variant is characterized, single-amplicon-based Sanger sequencing of a targeted viral genome segment is a cost-effective and quick alternative to variant tracking (19). Sanger sequencing can provide a larger read (~1.2 kb) to target a single amplicon of 0.5 to 1 kb, but samples such as saliva, nasal swabs, or even wastewater often lack intact RNA segments, making it challenging to generate larger amplicons in reverse transcriptase quantitative PCR (RT-qPCR)-based approaches. Based upon unique mutation signatures obtained from whole-genome sequence alignments of different SARS-CoV-2 variants and literature (WHO and CDC), shorter segments (<275 bp) of the spike protein receptor-binding domain were targeted for Sanger sequencing to identify variants quickly (Fig. 1).

## RESULTS

### RT-qPCR screening of saliva and nasal swab samples showed *C_T_* values ranging from 19 to 35.7.

Thirteen randomly selected positive saliva samples collected between February and October 2021 exhibited threshold cycle (*C_T_*) values ranging from 19.0 to 35.7 (Table 1; see also Fig. S1 in the supplemental material) and were considered positive samples for downstream Sanger sequencing. Six nasal swab samples received during the second week of December 2021 and one nasal swab sample received in the first week of February 2022 showed *C_T_* values ranging from 22.4 to 29.6 and were selected as positive samples for the Sanger sequencing process (Table 2; Fig. S2).

**TABLE 1:** Prescreening of saliva samples for COVID-19 using CDC N1 oligonucleotide pair

Sample	Date of collection (mo.day.yr)	*C_T_* value	Remark
Saliva 1	09.09.21	27.8	Positive
Saliva 2	09.15.21	31.4	Positive
Saliva 3	09.17.21	27.0	Positive
Saliva 4	09.22.21	24.0	Positive
Saliva 5	09.24.21	28.8	Positive
Saliva 6	09.29.21	31.5	Positive
Saliva 7	10.11.21	35.7	Positive
Saliva 8	04.27.21	23.8	Positive
Saliva 9	03.09.21	21.8	Positive
Saliva 10	03.09.21	19.0	Positive
Saliva 11	03.09.21	20.7	Positive
Saliva 12	03.05.21	27.4	Positive
Saliva 13	02.26.21	30.2	Positive

**TABLE 2:** Prescreening of nasal swabs for COVID-19 using CDC N1 oligonucleotide pair

Sample	Date of collection (mo.day.yr)	*C_T_* value	Remark
Nasal swab 1	12.11.21	24.0	Positive
Nasal swab 2	12.13.21	29.6	Positive
Nasal swab 3	12.13.21	27.3	Positive
Nasal swab 4	12.13.21	23.6	Positive
Nasal swab 5	12.13.21	22.4	Positive
Nasal swab 6	12.13.21	23.8	Positive
Nasal swab 7	02.05.22	22.6	Positive

### RBM-targeted Sanger sequencing identified SARS-CoV-2 variants of concern.

**FIG 2:**
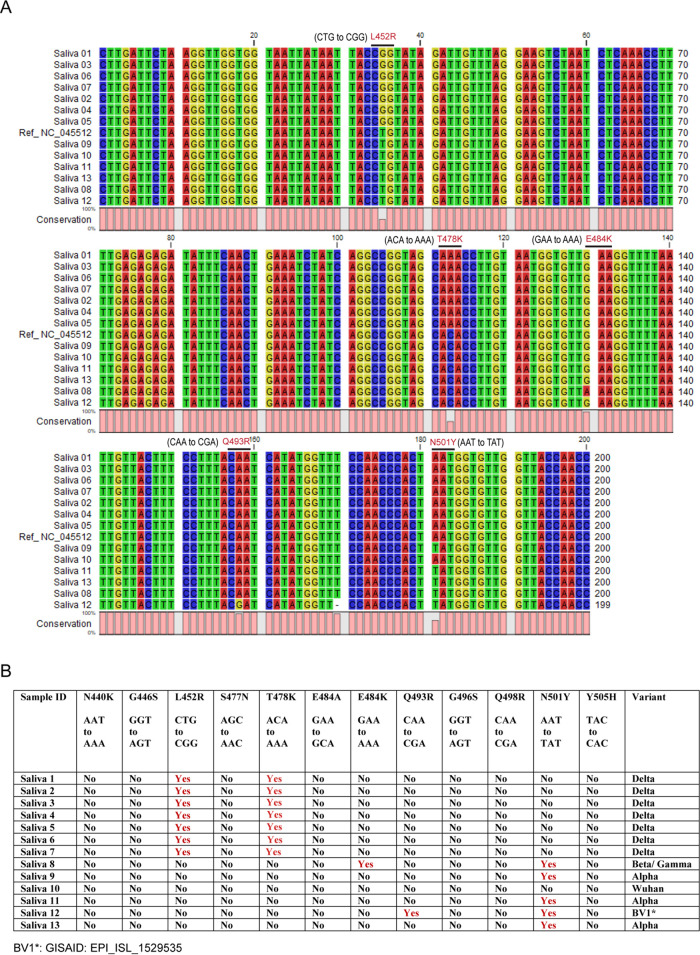
Sanger sequence alignment and variant detection of SARS-CoV-2 in saliva samples. (A) Sanger sequences obtained from the 246-bp amplicon of SARS-CoV-2 RBD are aligned against Wuhan reference sequence NC_045512 using CLC Sequence Viewer 8, and unique mutations (L452R, T478K, E484K, Q493R, and N501) are identified. (B) Based on the mutation profile, saliva samples are classified under Alpha, Beta/Gamma, Delta, or BV1 variants.

**FIG 3:**
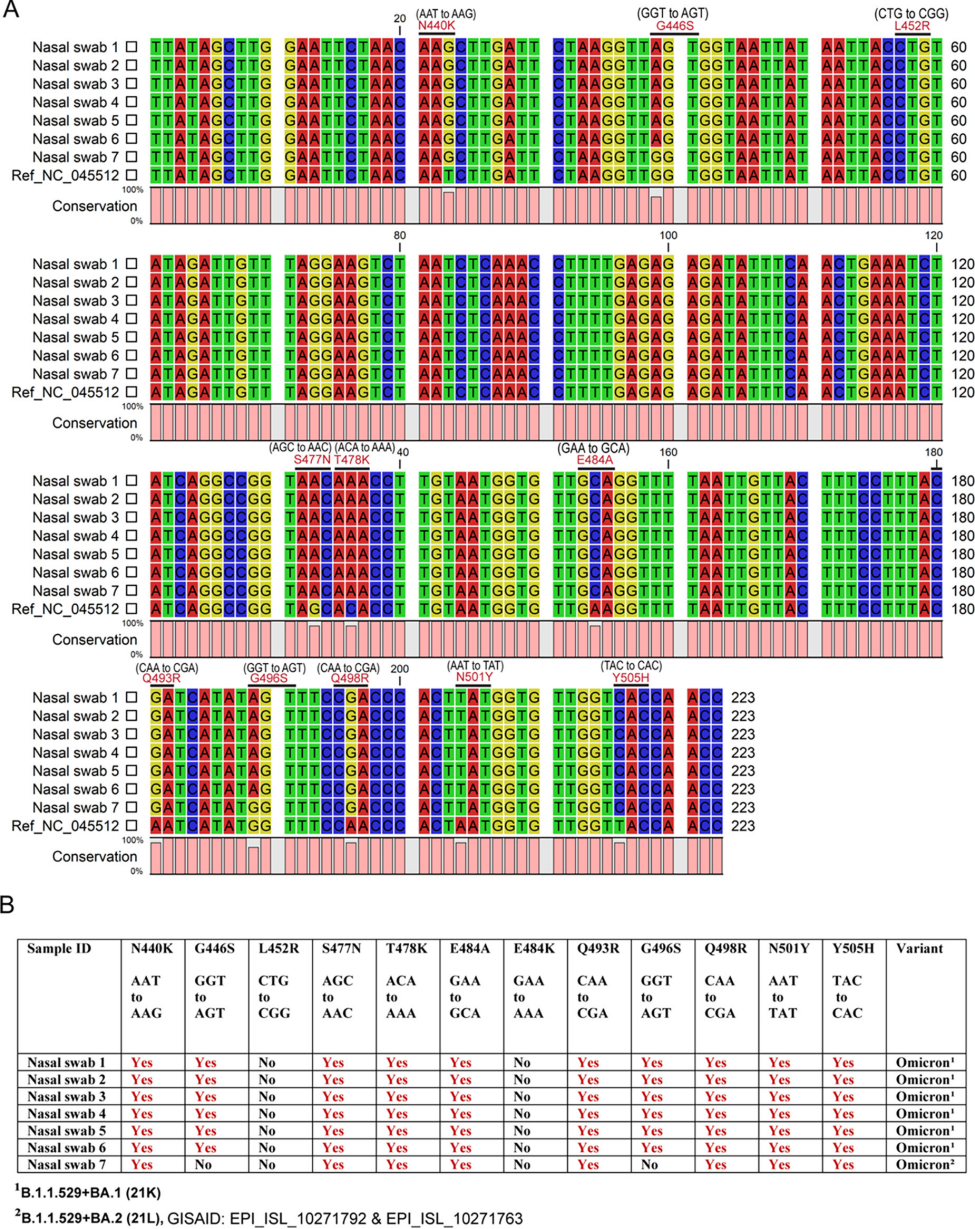
Sanger sequence alignment and variant detection of SARS-CoV-2 in nasal swab samples. (A) Sanger sequences obtained from the 273-bp amplicon of SARS-CoV-2 RBD are aligned against Wuhan reference sequence NC_045512 using the CLC Sequence Viewer, and unique mutations (N440K, G446S, S477N, T478K, E484A, Q493R, G496S, Q498R, N501Y, and Y505H) are identified. (B) Nasal swab samples are classified under Omicron variant and lineage based on mutation profile. Notably, G446S and G496S mutations in Omicron BA.1 are missing in Omicron BA.2 and confirmed by whole-genome sequencing.

Sanger sequencing from a gel-extracted PCR amplicon provided a clean chromatogram for reliable data analysis (Fig. S3 and S4). The use of a reverse primer for sequencing covered more mutation areas present in Sars-CoV-2 variants. The reverse-complemented sequencing data were used for sequence alignment and mutation analysis against SARS-CoV-2 Wuhan reference sequence NC_045512. Sequence alignment of the 246-bp amplicon revealed five different mutations in 13 saliva samples: L452R, T478K, E484K, Q493R, and N501Y (Fig. 2). Based on the presence or absence of a specific mutation(s), samples are designated under Alpha, Beta/Gamma, or Delta variants (Fig. 2; Fig. S3). Sequence alignment of the 273-bp amplicon revealed 10 mutations including N440K, G446S, S477N, T478K, E484A, Q493R, G496S, Q498R, N501Y, and Y505H in six nasal swab samples. These samples lack L452R and E484K mutations and are identified as Omicron BA.1 (B.1.1.529+BA.1). One nasal swab sample received on 5 February 2022 showed eight mutations including N440K, S477N, T478K, E484A, Q493R, Q498R, N501Y, and Y505H. This sample lacks G446S, L452R, E484K, and G496S mutations and is identified as Omicron BA.2 (B.1.1.529+BA.2) (Fig. 3; Fig. S4). Altogether, 12 mutations were detected in the receptor-binding motif across the 20 samples. These mutations are N440K, G446S, L452R, S477N, T478K, E484A, E484K, Q493R, G496S, Q498R, N501Y, and Y505H.

### The 273-bp RBD amplicon is capable of identifying 265 global occurrences of spike mutations.

**FIG 4:**
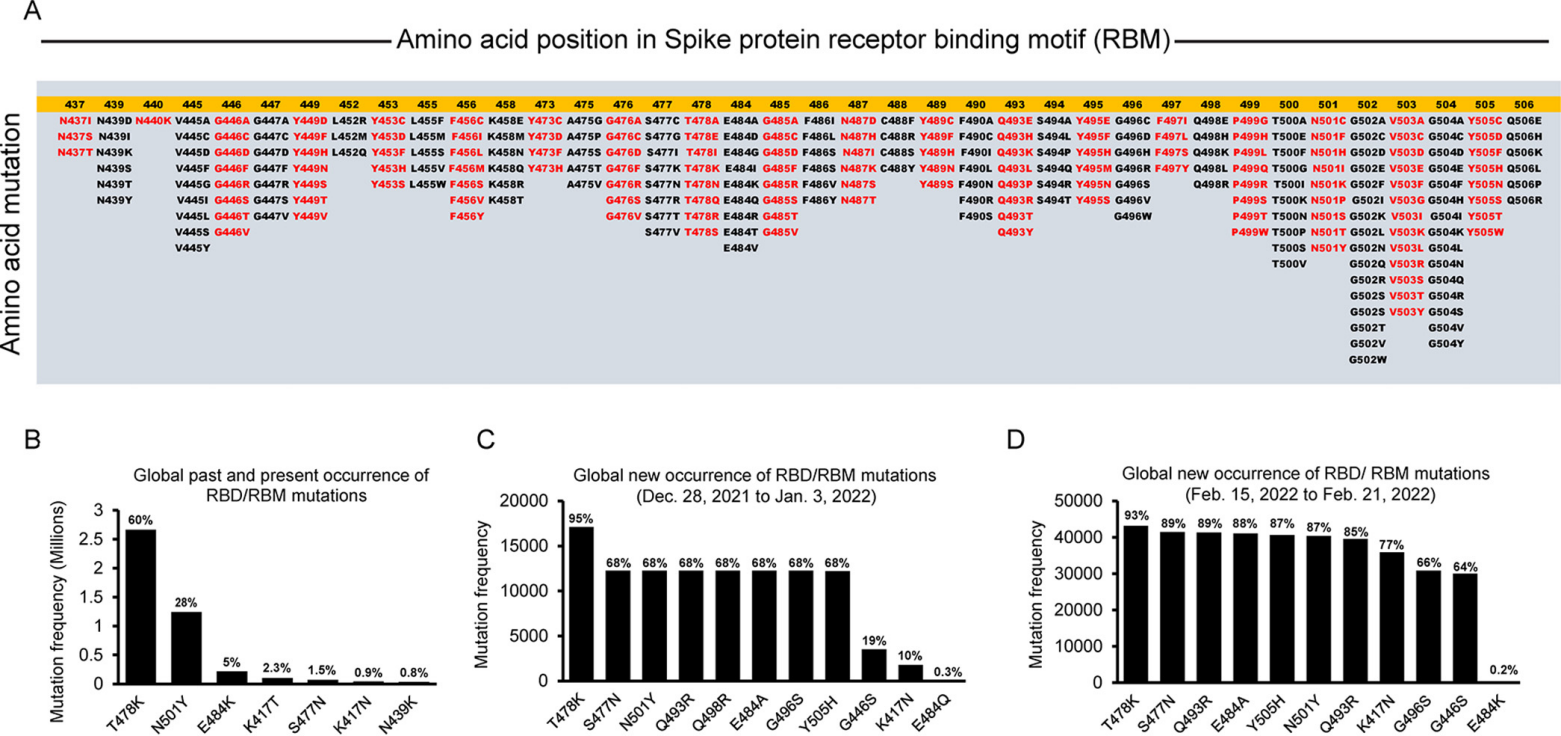
SARS-CoV-2 receptor-binding motif global mutation profile analyzed from GISAID surveillance data as of 21 February 2022. (A) Representative image showing 265 amino acid mutational changes in the receptor-binding motif of SARS-CoV-2 that occurred globally from October 2020 to February 2022. (B) A representative bar diagram shows global past and present occurrences of RBD/RBM mutations. The top seven amino acid mutational changes are shown. (C) A representative bar diagram of 18,037 genomes shows recent global occurrences of RBD/RBM mutations between 28 December 2021 and 3 January 2022. The top 11 amino acid mutational changes are shown. (D) A representative bar diagram shows recent global occurrences of RBD/RBM mutations between 15 and 21 February 2022. The top 11 amino acid mutational changes are shown.

In order to find the mutation detection capacity of the 273-bp RBD amplicon (229 bp excluding primer sequences), GISAID data on the global occurrence of mutations in the spike protein RBD as of 21 February 2022 were analyzed. The targeted 273-bp region of RBD covered 265 mutations that occurred globally and encompassed the whole region of RBM (438 to 506 amino acids [aa]) (Fig. 4A). When the past and present occurrences of RBD mutations were analyzed, 60% represented T478K, 28% represented N501Y, 5% represented E484K, 2.3% represented K417T, 1.5% represented S477N, 0.9% represented K417N, and 0.8% represented the N439K mutation (Fig. 4B). Interestingly, the global new occurrence of RBD mutation data from 28 December 2021 to 3 January 2022 revealed a T478K mutation occurrence of 95%; S477N, N501Y, Q493R, E484A, G496S, and Y505H mutation occurrences of 68%; a G446S mutation occurrence of 19%; a K417N mutation occurrence of 10%; and an E484Q mutation occurrence of 0.3% (Fig. 4C). We also observed some changes in amino acid mutation frequencies, particularly of Q498R, G446S, and K417N, in the third week of February 2022 (Fig. 4C and D). Except for amino acid position 417, all other major amino acid mutation points are covered under our study’s 273-bp targeted region.

## DISCUSSION

This study demonstrated an alternate strategy of SARS-CoV-2 variant identification in a timely and cost-effective manner, particularly for locations where WGS may not be possible. SARS-CoV-2, the causative agent of the COVID-19 pandemic, accounted for 430 million confirmed cases and 5.9 million deaths as of 21 February 2022 (1). The use of masks, social distancing, and vaccination measures are important in controlling the disease spread globally. However, frequent mutations with the new variant formation of SARS-CoV-2 make it difficult to control the pandemic. Therefore, early detection of viral mutations and overall variant surveillance are crucial as a countermeasure, such as preventive vaccine modification and therapeutic drug developments. Currently, WGS is mainly utilized for SARS-CoV-2 genome characterization and variant identification. However, this technique does have limitations as it involves significant equipment and reagent resources, personnel time, and, overall, higher costs. With the advancement of NGS technology, it is possible to reduce the sequencing cost and time significantly (20). However, reduced cost requires large sample numbers and equipment, which can impact diagnostic or clinical use or use as a surveillance tool. On the other hand, Sanger sequencing of prescreened positive samples with targeted amplification of a specific region of interest is a quick and cost-effective ($4 to $8 per single sample commercially) alternative for mutation analysis and SARS-CoV-2 variant tracking (19). In addition, samples with higher *C_T_* values (>30) often fail library preparation for whole-genome sequencing, making single amplicon-based Sanger sequencing a reasonable alternative (11, 21).

Initial screening of viral presence in saliva and nasal swabs in viral transport medium (VTM) precedes the sequencing process. It could also be used for other sample types, including urine, other body fluids, and water (22). In this study, CDC N1 oligonucleotide (23) pairs with 6-carboxyfluorescein (FAM) probe were utilized in RT-qPCR to screen samples for the presence of SARS-CoV-2 (see Fig. S1 in the supplemental material). Although the very recently identified Omicron variant showed a single nucleotide mutation in the 5′ end of the CDC N1 probe, this mutation did not impact RT-qPCR amplification in this study (Fig. S2).

The 30-kb single-stranded RNA of SARS-CoV-2 codes for 16 nonstructural proteins (NSP 1 to 16) and four structural proteins: spike (S), envelope (E), membrane (M), and nucleocapsid (N) (24). The receptor-binding domain (RBD), a fragment of ~194 amino acid residues (331 to 524 aa) (25) in the S1 subunit of spike protein, binds to the host cell ACE2 receptor (26), initiating S2 subunit unsheathing and membrane fusion (27). Within the RBD, an RBM contain 69 amino acid residues (438 to 506 aa) (28–30) overlapping the ACE2 binding site is immunodominant (31) and contains the majority of neutralizing epitopes (32). Nineteen of the 20 most potent neutralizing monoclonal antibodies (MAbs) have been mapped recently to the ACE2 binding site (33). The RBM region has more amino acid variability between SARS-CoV-2 and SARS-CoV (29). Mutational changes in the RBM region may impact virus transmissibility, antigenicity, and vaccine efficacy (34, 35). Therefore, the area of the SARS-CoV-2 RBD encompassing the RBM segment was targeted for PCR amplification and Sanger sequencing. The first primer pair was designed to amplify a 246-bp segment (440 to 522 aa) of the SARS-CoV-2 spike protein RBD (319 to 541 aa) and used to differentiate Alpha, BV1 (36), Beta/Gamma, and Delta variants. However, with the recent arrival of the Omicron variant and due to the presence of overlapping mutation points in the forward primer sequence, a new primer pair targeting a 273-bp segment (430 to 521 aa) of the RBD encompassing the whole area of the spike protein RBM (438 to 506 aa) was designed. This new primer pair did not show any mutation when aligned with past or present variants of concerns, including Omicron (Fig. S5A). The primer pair for both the 246-bp and 273-bp amplicons is also specific to SARS-CoV-2 as other coronaviruses showed several nucleotide mismatches (Fig. S5B). SARS-CoV-2 variant analysis of the samples with a targeted 273-bp RBD segment revealed 12 amino acid mutations across Alpha, Beta, Gamma, Delta, and Omicron variants and is ideal for quick variant surveillance. Variants identified in the samples matched the time frame of samples received versus existing variants of concern in the region. Samples received in February and March 2021 were dominated by the Alpha variant, while samples received in September showed the Delta variant (Table 1 and Fig. 2). Importantly, targeting the 273-bp RBD segment, the Omicron variant was identified in six samples received during the second week of December 2021 (Table 2 and Fig. 3). One nasal swab was received on 5 February 2022 (Table 2) and was identified as Omicron BA.2 (Fig. 3).

SARS-CoV-2 is continuously mutating, and it was reasonable to analyze the past and present global mutational changes in the spike protein RBD using GISAID data. Analysis revealed 265 mutations covering the whole area of the receptor-binding motif of SARS-CoV-2 spike protein (438 to 506 aa). Compared to the 246-bp region, the 273-bp region of the spike protein RBD is preferable and recommended for use in Sanger sequencing to identify the SARS-CoV-2 variants of past, present, and future concerns. Importantly, the 273-bp region can capture the unique L452Q mutation in the Omicron BA.2.12.1 lineage (e.g., GISAID no. EPI_ISL_12331770, submitted by Dakota Tyler Howard). Similarly, we can capture the L452R+F486V mutations in the recently found Omicron BA.4 and BA.5 lineages (e.g., GISAID no. EPI_ISL_12307641 and no. EPI_ISL_12307685, submitted by Daniel Gyamfi Amoako). To capture other subvariants of Omicron (BA.1, BA.1.1, BA.2, and BA.3), we have designed another pair of primers (F, 5′-ACAAACTTGTGCCCTTTTG-3′; R, 5′-TCATTTAATTTAGTAGGAGACACTCCA-3′) targeting a 170-bp region of RBD (333 to 389 aa), upstream of the 273-bp segment. This region can capture the unique R346K mutation in Omicron BA.1.1 (37) and the T376A mutation in Omicron BA.2 (38) lineages. Any new deletion, insertion, or mutational changes (if observed in the targeted sequencing region of spike protein RBD), although predictable for the new variant, must be confirmed by whole-genome sequencing. In this study, we identified our first Omicron BA.2 lineage from a nasal swab sample (nasal swab 7) using our 273-bp targeted Sanger sequencing (Fig. 3; Fig. S4), which was confirmed by whole-genome sequencing (GISAID: EPI_ISL_10271792, EPI_ISL_10271763).

### Conclusions.

Genomic surveillance of the SARS-CoV-2 variant is essential for vaccine modification or therapeutic development to combat the COVID-19 pandemic. Single-amplicon-based Sanger sequencing is a quicker and more cost-effective alternative for variant surveillance than whole-genome sequencing. This study demonstrated that RBM-targeted single-amplicon-based Sanger sequencing rapidly differentiated Alpha, Beta/Gamma, Delta, and Omicron variants in a cost-effective ($5/sample) way that can be adopted in a resource-limited setup. Importantly, targeted sequencing of the 170-bp and 273-bp spike protein regions provides an innovative way of molecular surveillance of currently circulating Omicron subvariants to counteract the disease spread.

## MATERIALS AND METHODS

### The strategy of the workflow.

Deidentified saliva or nasal swab samples in viral transport medium (VTM) were received from a local COVID-19 surveillance screening program. Samples were screened via RT-qPCR for the presence of the SARS-CoV-2 N1 gene region to identify positive samples. Viral RNA was isolated from positive samples and used for RBM-targeted short amplicon generation, gel purification, and Sanger sequencing. Finally, variants were identified and assigned based on their respective mutation signatures (Fig. 1).

### Primer design for Sanger sequencing to capture unique mutations in SARS-CoV-2 variants.

Reference sequences of SARS-CoV-2 variants were obtained from online databases, including those of the Global Initiative on Sharing All Influenza Data (GISAID) and the National Center for Biotechnology Information (NCBI). Primer pairs for Sanger sequencing were designed based upon the SARS-CoV-2 spike protein sequence mutational characteristics of previous up to the most recent variants of concern. Considering viral instability and nucleic acid fragmentation in various samples, including saliva, nasal swabs, or wastewater samples, a smaller vulnerable segment of 246 bp (RBD-F, 5′-TCTTGATTCTAAGGTTGGTGGT-3′, and RBD-R, 5′-GCTGGTGCATGTAGAAGTTCA-3′) or 273 bp (RBD-F, 5′-AGGCTGCGTTATAGCTTGGA-3′, and RBD-R, 5′-GGTGCATGTAGAAGTTCAAAAGAA-3′) that encompasses the receptor-binding motif was targeted. Primer sequences were aligned with SARS-CoV-2 variants of concern as well as with other coronavirus sequences for specificity (see Fig. S5 in the supplemental material). The forward oligonucleotide for the 246-bp amplicon showed two nucleotide mutation areas corresponding with the known Omicron sequence. Importantly, no mutation area was found between SARS-CoV-2 variants and the oligonucleotide pair for the 273-bp amplicon. Primers for both 246-bp and 273-bp amplicons are specific to SARS-CoV-2 compared with other coronavirus nucleotide sequences (Fig. S5).

### RT-qPCR screening for SARS-CoV-2.

Deidentified saliva and nasal swab samples were heat inactivated (50 μL of sample was diluted [1:1] in 50 μL of 2× Tris-borate-EDTA [TBE] containing 1% Tween 20 and heated at 95°C for 15 min) and then analyzed for the presence of SARS-CoV-2 under biosafety level 2 conditions. RT-qPCR screening was performed using the CDC N1 oligonucleotide pair/FAM probe (CDC N1-F, 5′-GACCCCAAAATCAGCGAAAT-3′; CDC N1-R, 5′-TCTGGTTACTGCCAGTTGAATCTG-3′; and Probe CDC N1, 5′ FAM-ACCCCGCATTACGTTTGGTGGACC-BHQ1 3′) and the Luna Universal Probe one-step RT-qPCR kit (catalog no. E3006; NEB, Ipswich, MA, USA). A 20-μL RT-qPCR mixture contained 7 μL of sample, 0.8 μL each of forward and reverse oligonucleotides (10 μM), 0.4 μL of probe (10 μM), and 11 μL of NEB Luna 2× master mix. PCR cycle steps followed incubation at 55°C for 10 min (1 cycle of cDNA synthesis), 95°C for 1 min (1 cycle), and 95°C for 10 s and 60°C for 30 s (41 cycles). Crossing threshold (*C_T_*) values of <38 were considered presumptively positive and sent to a CLIA diagnostic laboratory for confirmation. *C_T_* values between 38 and 40 were considered indeterminate and were also sent to the diagnostic laboratory for an official determination. All positive samples were stored at −80°C for RNA extraction and sequencing.

### Viral RNA extraction from selected positive samples.

Viral RNA was isolated from randomly selected and prescreened positive samples (from either saliva or nasal swab in VTM) using the Monarch total RNA miniprep kit (catalog no. T2010S; NEB, Ipswich, MA, USA) according to the manufacturer’s instruction. In brief, 200 μL of sample was mixed with 200 μL of DNA/RNA protection reagent and incubated with 5 μL of proteinase K (20 mg/mL) for 15 min at room temperature. Samples were lysed with 800 μL of RNA lysis buffer and then passed through a DNA column to capture and remove any residual DNA. RNA was precipitated in eluted samples using 600 mL of isopropanol and captured in the RNA column. After washing and drying the column, RNA was eluted in 50 μL of nuclease-free water and stored at −80°C.

### cDNA synthesis, PCR amplification, gel purification, and Sanger sequencing.

cDNA was synthesized from 6 μL of RNA using the SuperScript IV first-strand synthesis system (catalog no. 18091050; Invitrogen, CA, USA). The resulting cDNA served as the template for subsequent region-targeted PCR amplification for sequencing. Briefly, 3 μL of cDNA was included in a 50-μL GoTaq Green master mix reaction mixture (catalog no. M7123; Promega, Madison, WI, USA) containing 5 μL of forward and reverse oligonucleotide (10 μM) and amplified via PCR according to the manufacturer’s instructions. The amplified product (246 bp or 273 bp) was resolved in a 1.5% agarose gel, the gel band was excised, and the nucleic acid fragment was purified from the gel band using a spin column. Alternatively, the PCR product can be column purified in lieu of gel purification. Once purified, 8 μL (100 to 250 ng) of purified DNA was mixed with 2 μL of 10 μM oligonucleotide (preferably reverse oligonucleotide), packaged for shipping, and sent to a commercial Sanger sequencing service provider (Eurofins Genomics, Louisville, KY, USA). The sequencing results were typically received within 24 to 72 h. The sequence data were analyzed using CLC Sequence Viewer 8 (CLC bio LLC, Cambridge, MA, USA) for mutation analysis and variant identification.

### Whole-genome sequencing of SARS-CoV-2.

Whole-genome sequencing for SARS-CoV-2 was carried out to confirm variants. Library preparation was performed using the Swift SNAP SARS-CoV-2 amplicon panel and then sequencing using Illumina NextSeq 550. Approximately 1 million reads per sample were obtained, mapped, and assembled using BWA. Available sequences were deposited in GISAID (see below).

### GISAID data analysis of the global occurrence of RBM mutations.

GISAID human CoV-19 (hCoV-19) spike glycoprotein mutation surveillance data from 4,454,682 complete genomes were analyzed as of 21 February 2022. The frequency of past and present amino acid changes in RBM for the 4,454,682 complete genomes and global new occurrences of RBM mutations for 18,037 and 46,590 new complete genomes were analyzed. Amino acid changes overlapping the amino acid position in the spike protein receptor-binding motif were aligned with our targeted RBM region to find the past and present overlapping mutation points.

### Data availability.

We have included all the data associated with the article as figures and tables with the main text or as figures in the supplemental material. Whole-genome sequencing data are available in the GISAID database (https://www.gisaid.org/) under the following accession numbers: saliva 1, EPI_ISL_10707978 and EPI_ISL_10707984; saliva 9, EPI_ISL_1626920; saliva 10, EPI_ISL_1626934; saliva 11, EPI_ISL_1626942; saliva 12, EPI_ISL_1529535; saliva 13, EPI_ISL_1626951; nasal swab 1, EPI_ISL_11044451; nasal swab 2, EPI_ISL_11044452; nasal swab 3, EPI_ISL_11044453; nasal swab 5, EPI_ISL_11044454; nasal swab 6, EPI_ISL_11044455; nasal swab 7, EPI_ISL_10271763 and EPI_ISL_10271792.
