# Supplementary material for: Receptor-Binding-Motif-Targeted Sanger Sequencing: a Quick and Cost-Effective Strategy for Molecular Surveillance of SARS-CoV-2 Variants
Source: Microbiol Spectr. 2022 May 31;10(3):e00665-22. doi: 10.1128/spectrum.00665-22 (PMC9241651; doi:10.1128/spectrum.00665-22)
Supplement: SUPPLEMENTAL FILE 1 — Supplemental material. Download spectrum.00665-22-s0001.pdf, PDF file, 6.6 MB [file spectrum.00665-22-s0001.pdf]

Supplemental figure legends

Figure S1

Identification of SARS-CoV2 positive saliva samples using RT_qPCR. Diluted (1:1) and heat inactivated saliva samples are used to amplify SARS-CoV2 employing CDC N1 oligo and FAM-probe in a one step RT_qPCR. Representative chromatograms showing PCR amplifications of 13 positive saliva samples with CT values ranging from 19.0 to 35.7.

Figure S2

Identification of SARS-CoV2 positive nasal swab samples using RT_qPCR. Diluted (1:1) and heat inactivated nasal swab samples are used to amplify SARS-CoV2 employing CDC N1 oligo and FAM-probe in a one-step RT_qPCR. Representative chromatograms showing PCR amplifications of 6 positive nasal swab samples with CT values ranging from 22.4 to 29.6

Figure S3

Chromatograms of Sanger sequences obtained from saliva-isolated SARS-CoV2. A single PCR amplicon of 246 bp was generated from purified SARS-CoV2 RNA and used for Sanger sequencing using reverse oligo. This chromatogram corresponds with the data in Figure 2.

Figure S4

Chromatograms of Sanger sequences obtained from nasal swab-isolated SARS-CoV2. A single PCR amplicon of 273 bp was generated from purified SARS-CoV2 RNA and used for Sanger sequencing using reverse oligo. This chromatogram corresponds with the data in Figure 3.

Figure S5.

Oligo alignment with SARS-CoV2 and other corona virus sequences. (A) SARS-CoV2 sequence alignment revealed two nucleotide mismatches (rectangle) with the forward oligo for 246 bp amplicon vs. Omicron variant (rectangle: T/G and G/A). Oligo pair sequences for the 273 bp amplicon aligned perfectly with all of the corresponding regions of SARS-CoV2 variants. (B) Corona virus sequence alignment reveals that the oligos for both 246 bp and 273 bp amplicons are specific to SARS-CoV-2 as matches perfectly, but not with other corona virus sequences those showing several nucleotide mismatches.

Figure S1

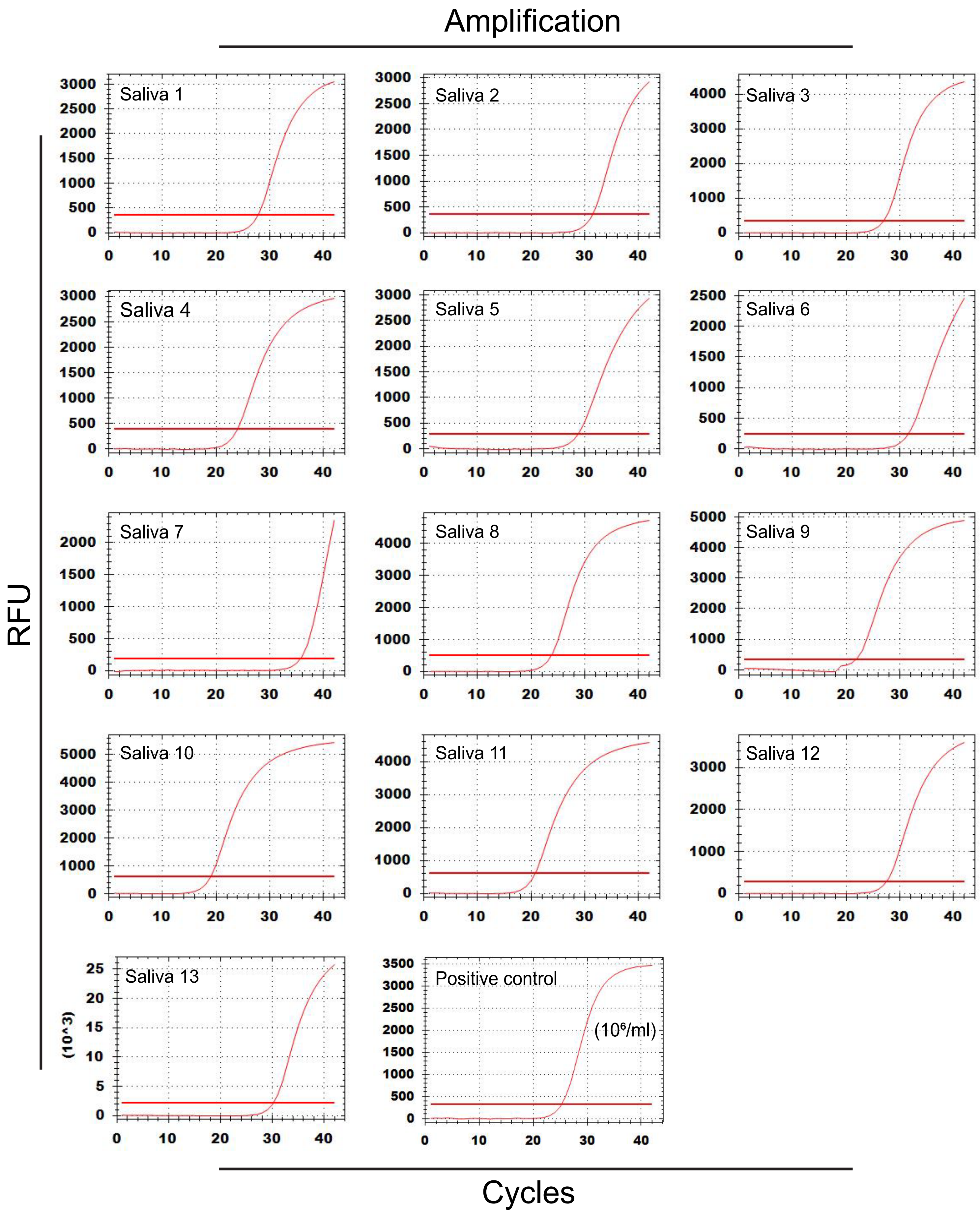
Amplification
RFU
Cycles
Saliva 1
Saliva 2
Saliva 3
Saliva 4
Saliva 5
Saliva 6
Saliva 7
Saliva 8
Saliva 9
Saliva 10
Saliva 11
Saliva 12
Saliva 13
(10^3)
Positive control
(10⁶/ml)

Figure S2

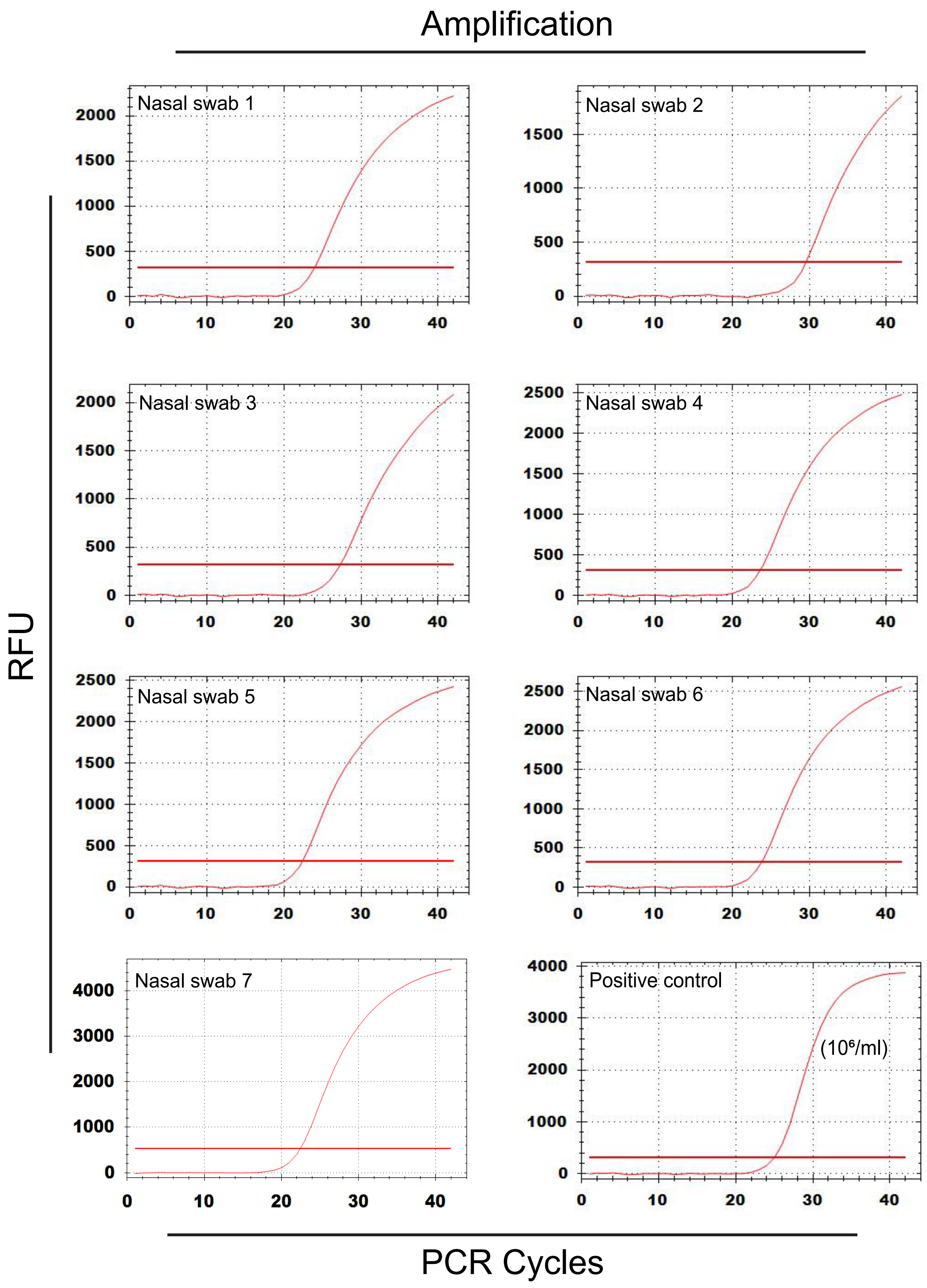

Amplification
RFU
Nasal swab 1
Nasal swab 2
Nasal swab 3
Nasal swab 4
Nasal swab 5
Nasal swab 6
Nasal swab 7
Positive control
(10⁶/ml)
PCR Cycles

Figure S3

Saliva 1

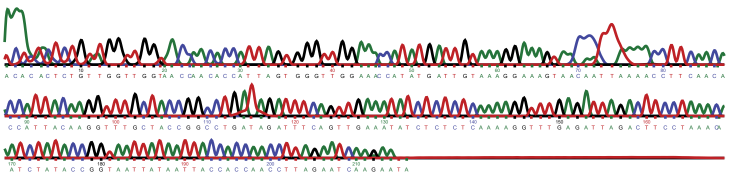

Saliva 2

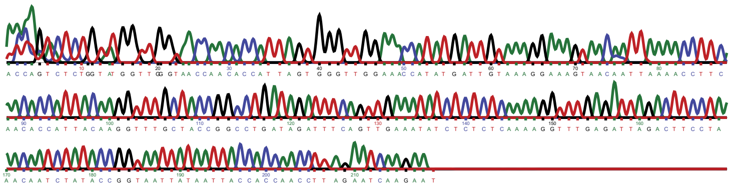

Saliva 3

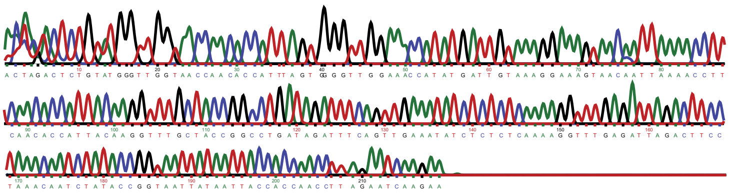

Saliva 4

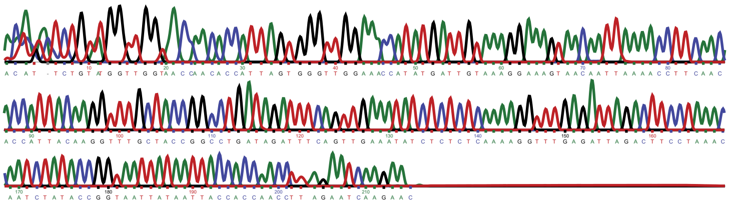

Saliva 5

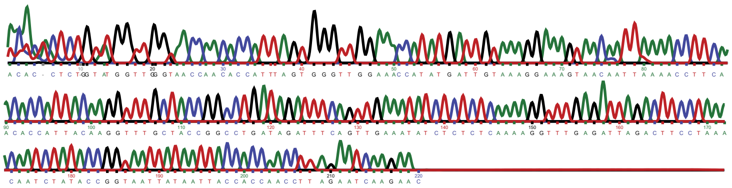

Saliva 6

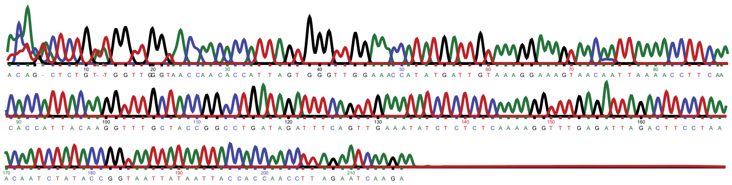

Saliva 7

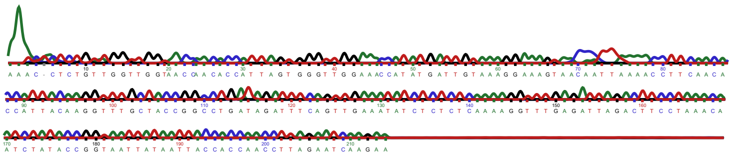

Saliva 8

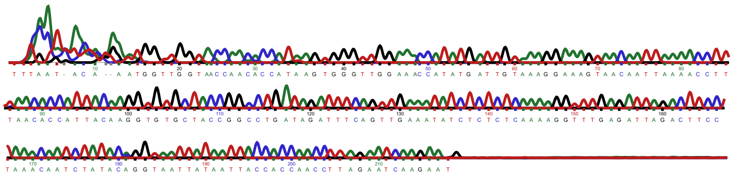

Saliva 9

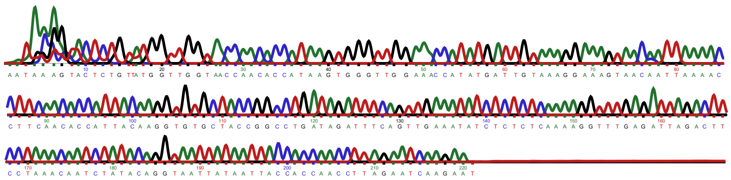

Saliva 10

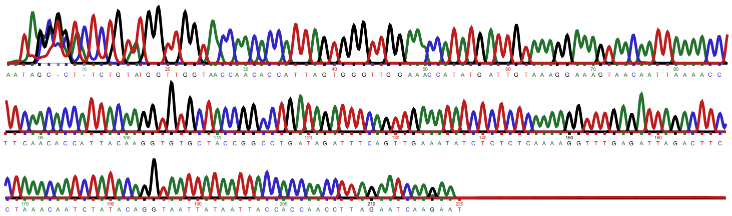

Saliva 11

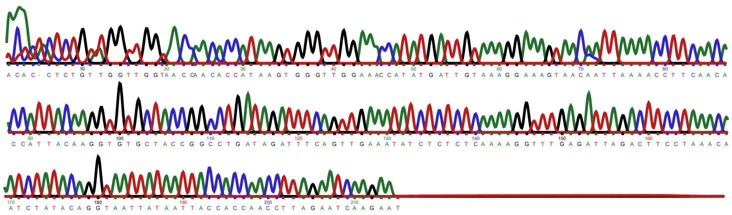

Saliva 12

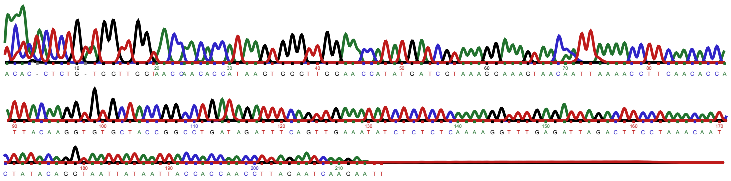

Saliva 13

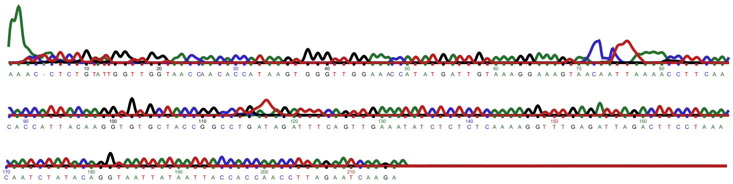

Figure S4

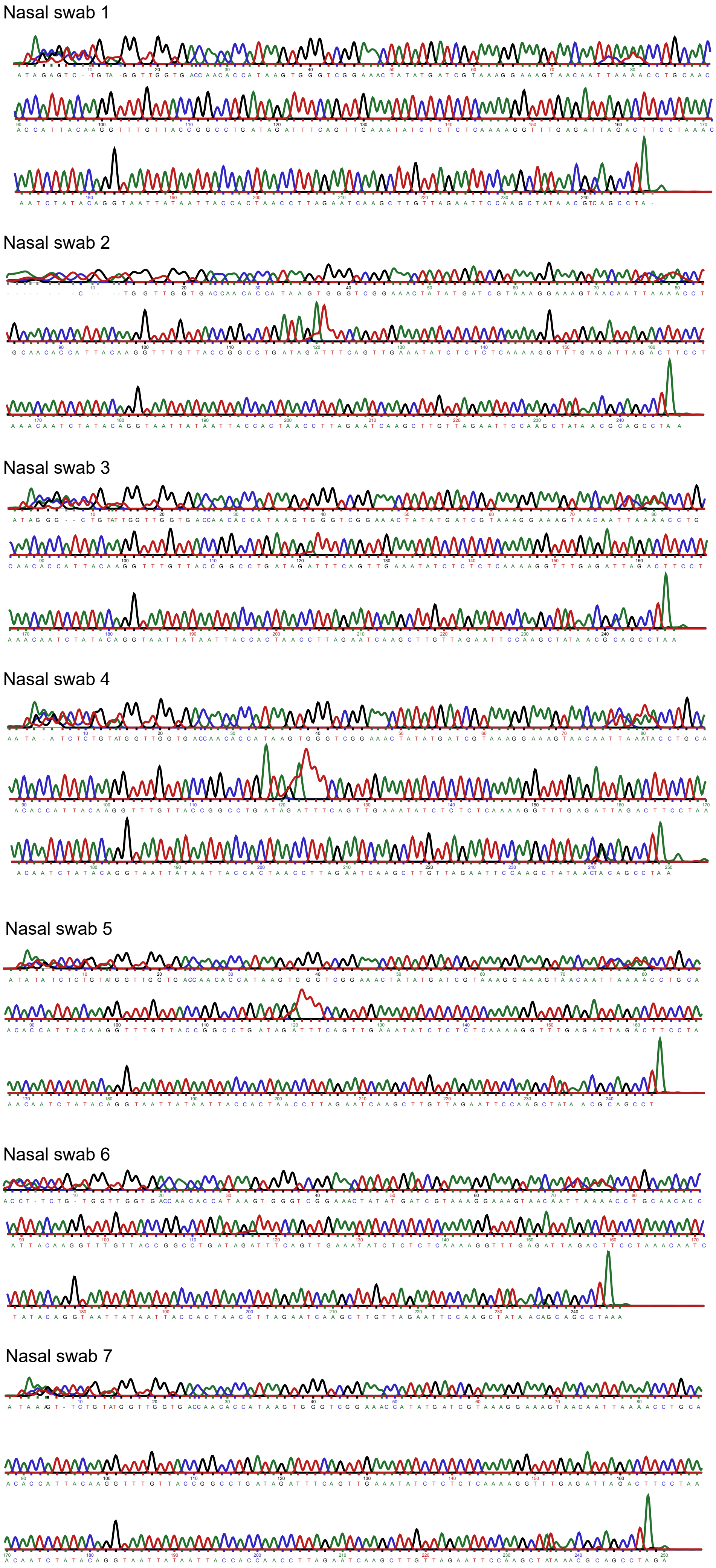
Nasal swab 1
Nasal swab 2
Nasal swab 3
Nasal swab 4
Nasal swab 5
Nasal swab 6
Nasal swab 7

Figure S5

A

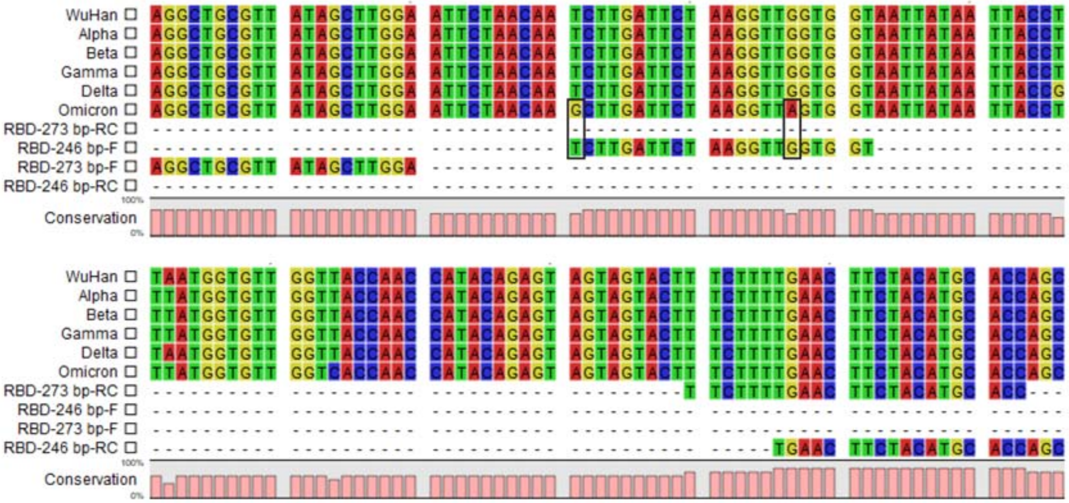

WuHan
Alpha
Beta
Gamma
Delta
Omicron
RBD-273 bp-RC
RBD-246 bp-F
RBD-273 bp-F
RBD-246 bp-RC
Conservation

B

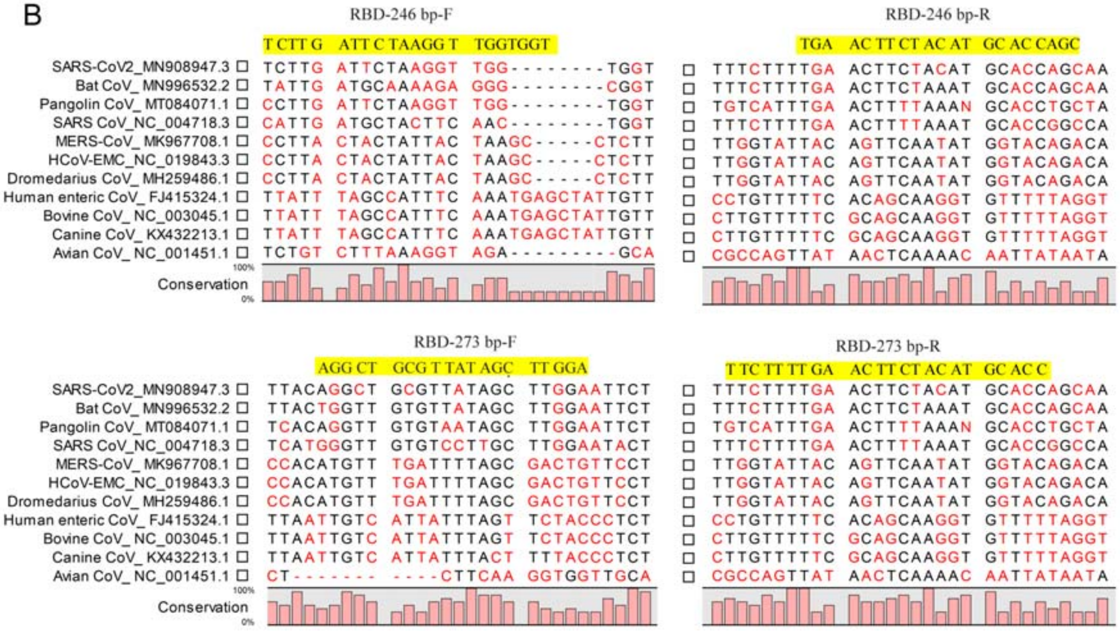

RBD-246 bp-F
RBD-246 bp-R
RBD-273 bp-F
RBD-273 bp-R
SARS-CoV2_MN908947.3
Bat CoV_ MN996532.2
Pangolin CoV_ MT084071.1
SARS CoV_NC_004718.3
MERS-CoV_ MK967708.1
HCoV-EMC_NC_019843.3
Dromedarius CoV_ MH259486.1
Human enteric CoV_ FJ415324.1
Bovine CoV_ NC_003045.1
Canine CoV_ KX432213.1
Avian CoV_ NC_001451.1
Conservation

Table S1. GISAID Accession Numbers of whole genome sequencing.

Sample	GISAID Accession Numbers
Saliva 1	EPI_ISL_10707978, EPI_ISL_10707984
Saliva 9	EPI_ISL_1626920
Saliva 10	EPI_ISL_1626934
Saliva 11	EPI_ISL_1626942
Saliva 12	EPI_ISL_1529535
Saliva 13	EPI_ISL_1626951
Nasal swab 1	EPI_ISL_11044451
Nasal swab 2	EPI_ISL_11044452
Nasal swab 3	EPI_ISL_11044453
Nasal swab 5	EPI_ISL_11044454
Nasal swab 6	EPI_ISL_11044455
Nasal swab 7	EPI_ISL_10271763, EPI_ISL_10271792
